# Transition Probabilities of Noise-induced Transitions of the Atlantic Ocean Circulation

**DOI:** 10.1038/s41598-019-56435-6

**Published:** 2019-12-30

**Authors:** Daniele Castellana, Sven Baars, Fred W. Wubs, Henk A. Dijkstra

**Affiliations:** 1 Institute for Marine and Atmospheric research Utrecht, Department of Physics, Utrecht University, Utrecht, The Netherlands; 2 Bernoulli Institute for Mathematics, Computer Science and Artificial Intelligence, University of Groningen, Groningen, The Netherlands; 3 Centre for Complex Systems Studies, Department of Physics, Utrecht University, Utrecht, The Netherlands

**Keywords:** Climate sciences, Ocean sciences, Applied mathematics

## Abstract

The Atlantic Meridional Overturning Circulation (AMOC) is considered to be a tipping element of the climate system. As it cannot be excluded that the AMOC is in a multiple regime, transitions can occur due to atmospheric noise between the present-day state and a weaker AMOC state. For the first time, we here determine estimates of the transition probability of noise-induced transitions of the AMOC, within a certain time period, using a methodology from large deviation theory. We find that there are two types of transitions, with a partial or full collapse of the AMOC, having different transition probabilities. For the present-day state, we estimate the transition probability of the partial collapse over the next 100 years to be about 15%, with a high sensitivity of this probability to the surface freshwater noise amplitude.

## Introduction

The Atlantic Ocean circulation has a special role in the climate system, because its associated meridional heat transport is positive over all latitudes^1^, peaking at about 1.5 PW at 26.5°N^2^. Responsible for this heat transport is the Atlantic Meridional Overturning Circulation (AMOC) which is the zonally integrated volume transport generated by a complex system of Atlantic Ocean currents. Over the period 2008–2012 the mean AMOC strength at 26.5°N decreased by about 2.7 Sv compared to the period 2004–2008, when it was about 17.5 Sv. There were also short intervals (e.g. at the end of 2009) where the AMOC strength was nearly zero^3^.

There is convincing evidence, both from observations and model results, that the strength of the AMOC is sensitive to the surface freshwater forcing^4,5^. About sixty years ago, this sensitivity was already studied in an idealized box model^6^ and it was shown that the dependence of the AMOC strength on both temperature and salinity (through the density) can lead to a multiple equilibria regime. In so-called Earth system Models of Intermediate Complexity (EMICs), such regimes have been detected through hysteresis behaviour of the AMOC once the surface freshwater strength is varied^7^. The multiple equilibria regime is then bounded by relatively sharp transitions between a strong and weak AMOC state.

Although such hysteresis behaviour has also been found^8^ in relatively low-resolution Global Climate Models (GCMs), the computations to detect multiple equilibria regimes in state-of-the-art GCMs are demanding and have not been carried out systematically. Nevertheless, the AMOC is listed among the ‘tipping elements’ of the Earth system^9^, because it can not be excluded that the present-day climate state is in a multiple equilibria regime. For this reason, EMICs have been used to develop indicators of the multiple equilibria AMOC regime, that can be easily computed from model results and the sparse instrumental record.

Following initial ideas by *Rahmstorf*^10^ and *de Vries and Weber*^11^, a well-known indicator is based on the AMOC-induced freshwater divergence in the Atlantic, referred to as ∑ in^12^, later reintroduced as Δ*M*_*ov*_ by *Liu et al.*^13,14^. The component at the southern boundary of the Atlantic (at 35°S) is much larger than the northern component (at 65°N) and is often referred to as *M*_*ov*_^11,15^ (or *F*_*ov*_^8^). A negative (positive) value of *M*_*ov*_ indicates that the AMOC is in a multiple (single) equilibria regime. *M*_*ov*_ was developed from ocean-only model results and hence neglects the effects of atmospheric feedbacks^16^. However, it has been widely used in models to interpret the behaviour of the AMOC^17,18^. Present-day observational results show that *M*_*ov*_ is in the range of −0.35 Sv to −0.1 Sv^13,15,19,20^. Hence, if *M*_*ov*_ is indeed a proper indicator, then the present-day AMOC is in a multiple equilibria regime.

In this case, a change in the surface freshwater forcing can induce a transition to a (weak) AMOC state, often referred to as bifurcation tipping. In addition, a relatively large perturbation in any observable may also induce such a transition, often referred to as noise-induced tipping. Indeed, paleoclimate studies have shown that abrupt changes in atmospheric temperatures associated with the Dansgaard-Oeschger cycles, which are thought to be closely related to changes in AMOC strength, may have been noise-induced^21,22^. However, to determine the probability of an AMOC transition within a certain period of time (e.g. into the future), for example due to noise in the surface freshwater forcing, is a difficult problem which has not been addressed yet.

Here, using techniques from large-deviation theory^23^, we present results for the transition probabilities of noise-induced changes in the AMOC. As such techniques are still computationally demanding, we apply them here to a detailed box model of the AMOC, in which the value of *M*_*ov*_ is a precise measure of the multiple equilibria regime^17^. By using *M*_*ov*_, we can make an estimate, from the model results, of the transition probabilities for the observed present-day AMOC.

## Model and Methods

**Figure 1:**
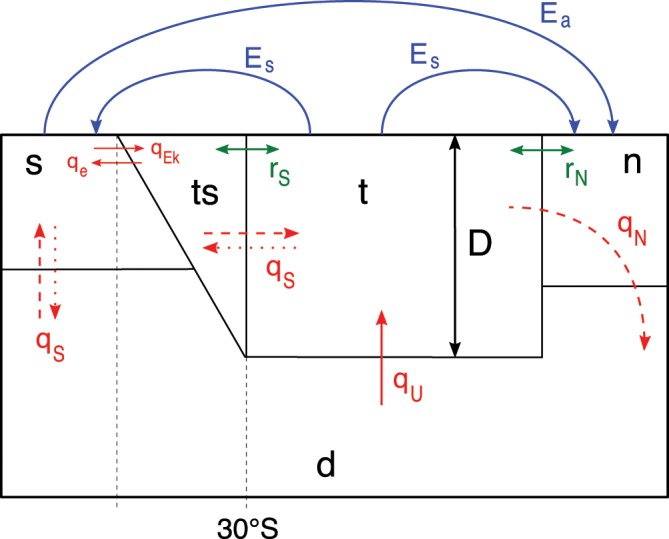
Sketch of the box model, adapted from *Cimatoribus et al.*^17^. The red, blue and green arrows represent, respectively, the volume fluxes between the boxes, the freshwater exchange between the basin and the atmosphere, and the effect of the subtropical gyres on the transport. The dashed (dotted) arrows indicate fluxes present only in the AMOC ‘on’ (‘off’) state: in the collapsed state, the downwelling (*q*_*N*_) stops and the transport *q*_*S*_ is reversed.

The model used is an extension of the one developed by ^*Cimatoribus et al.*17^ and represents the Atlantic Ocean as five boxes (Fig. 1). A deep box (labelled as *d*) extends throughout the whole latitudinal width of the basin. It is separated from the upper ocean layer by a pycnocline, whose depth *D* is a dependent quantity in the model. Two other boxes *s* and *n* represent, respectively, the Southern Ocean and the northern part of the Atlantic Ocean, where the downwelling of dense water takes place. The pycnocline layer is represented by two boxes (*t* and *ts*) where the latter is located south of 30°S. Thanks to this splitting, it is possible to distinguish between the freshwater transported by the AMOC and by the southern subtropical gyre circulation (more details are given in^17^).

Density variations in the boxes are solely due to the salinity variations, as the temperature in each box is assumed to be constant. The downwelling in the North Atlantic (and hence the AMOC strength) is represented by the term *q*_*N*_, while *q*_*S*_ is the difference between the wind-driven Ekman flow (*q*_*EK*_) and the eddy-induced flow (*q*_*e*_), the latter associated with baroclinic instabilities of the Antarctic Circumpolar Current. The upwelling volume transport from the deep layer to the pycnocline layer is represented by *q*_*U*_. Two coefficients, *r*_*N*_ and *r*_*S*_, capture the effect of the wind-driven subtropical gyres on the salinity transport. Finally, the Atlantic Ocean circulation model is forced by external freshwater fluxes, split into a symmetric (*E*_*s*_) and an asymmetric (*E*_*a*_) component (Fig. 1); the noise will only be incorporated through *E*_*a*_.

The equations determining the evolution of the AMOC in this model are the salinity budgets of the different boxes, together with the variation of the volume of the pycnocline, and the salt and volume conservation equations; these are given in the Supplementary Section A. The model in^17^ has been extended to allow for the existence of both a strong and weak AMOC state, which we refer to below as the ‘on’ and ‘off’ state, respectively. The present-day state of a strong AMOC is represented by values *q*_*N*_ > *q*_*S*_ > *q*_*U*_ > 0. The ‘off’ AMOC state occurs only when the downwelling in the Northern Atlantic stops (*q*_*N*_ = 0) and the circulation in the South Atlantic is reversed (*q*_*S*_ < 0).

The model is particularly suitable for investigating the variability of the AMOC due to the buoyancy anomalies. While capturing the essential physical processes involved in the dynamics of the AMOC (e.g. the wind-induced upwelling in the Southern Ocean), it gives emphasis to the salt-advection feedback, which is the feedback responsible for the collapse of the AMOC. In the real ocean, wind-induced variability can lead to the occurrence of extremes in the strength of the AMOC, as found in the RAPID measurements^24^. These effects are not captured by our model. Nevertheless, this does not affect the capabilities of the model to capture the dominant processes underlying the buoyancy-induced variability of the AMOC. Recently *Wood et al.*^25^ also showed that a 5-box dynamical model can be calibrated with GCM output to capture (within some error bars) the critical behaviour of the AMOC. In conclusion, the model used here, forced by the interannual time scale noise in the freshwater flux, is fit for purpose to look at freshwater-caused transitions (in a bistable AMOC regime) which are affected by the salt-advection feedback.

Transition probabilities between states in this model are computed using the Trajectory-Adaptive Multilevel Sampling (TAMS) method^23^. Estimating transition probabilities for a general stochastic system is a challenging task. Analytical results are available only for special classes of systems, whereas brute-force methods, consisting of simulating a large ensemble of trajectories and then counting the number of observed transitions, are not feasible when the probabilities involved are small.

The idea behind TAMS, which belong to the so-called rare event algorithms, is the following: the method tries to push trajectories into the direction of the destination equilibrium, to make sure that transitions actually occur (also for small sample sizes) while still being able to compute the transition probability. See Supplementary Section B for more information and an example.

## Results

Variations in the forcing will only be considered through the asymmetric freshwater flux, which is written as

1 $${E}_{a}(t)={\bar{E}}_{a}+{E}_{a}^{\sigma }(t).$$

here the deterministic value ${\bar{E}}_{a}$ is constant and the stochastic part (${E}_{a}^{\sigma }(t)=\sigma \zeta (t)$) is represented by a zero mean, unit variance white noise process *ζ*(*t*) and a standard deviation *σ*.

**Figure 2:**
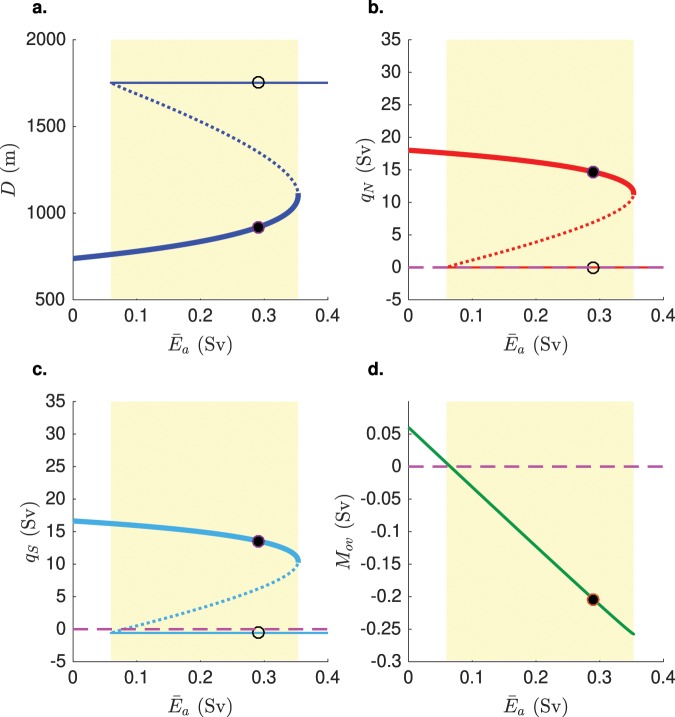
(**a**) Bifurcation diagram for the depth of the pycnocline versus the parameter ${\bar{E}}_{a}$. Solid (dashed) lines indicate stable (unstable) equilibria of the system. Among the solid lines, the thicker ones represent the AMOC ‘on’ state, while the thinner ones correspond to the ‘off’ state. The yellow area indicates the bistable regime. The black circles indicate the value of ${\bar{E}}_{a}$ chosen for the time simulation in Fig. 3. The starting (destination) equilibrium belongs to the ‘on’ (‘off’) branch of the diagram. (**b**,**c**) The same diagrams, respectively for the downwelling in the North Atlantic (*q*_*N*_) and the upwelling in the Southern Ocean (*q*_*S*_). The dashed magenta lines indicate null transport: in the collapsed state, the downwelling stops, while the circulation through the basin is reversed, as indicated by the negative value of *q*_*S*_. (**d**) *M*_*ov*_, calculated following Eq. (2), as a function of the asymmetric freshwater forcing ${\bar{E}}_{a}$. The indicator is calculated only for the ‘on’ state of the system. *M*_*ov*_ is positive (negative) in the monostable (bistable) regime of the AMOC.

Steady states of the deterministic model (*σ* = 0) for standard values of the parameters (see the Supplementary Table S1) are shown in Fig. 2. Two saddle-node bifurcation points bound the multiple equilibria regime: for each value of ${\bar{E}}_{a}$ in the range [0.06, 0.35], both ‘on’ and ‘off’ AMOC states exist (Fig. 2). The pycnocline depth is much shallower (up to 1000 m) for the ‘on’ AMOC state (Fig. 2a) than for the ‘off’ AMOC state. The main difference between the diagrams shown in Fig. 2 and the ones that can be found in^17^ is the presence of the ‘off’ AMOC state, since the model allows solutions with *q*_*N*_ = 0 (Fig. 2b) and *q*_*S*_ < 0 (Fig. 2c). The freshwater transport at 30°S carried by the AMOC is given by

2 $${M}_{ov}=-\,\frac{{q}_{S}}{{S}_{0}}({S}_{ts}-{S}_{d}).$$

As already shown in^17^, the sign of *M*_*ov*_ is an adequate indicator for the multiple equilibria regime (Fig. 2d). For further reference below, the bifurcation diagrams of the salinities in the different boxes and the upwelling strength *q*_*U*_ are shown in the Supplementary Section C, for example, showing a constant upwelling in the ‘off’ AMOC state.

We next fix the deterministic part of the asymmetric freshwater forcing (${\bar{E}}_{a}$) to a value within the multiple equilibria regime and add stochastic forcing (*σ* > 0). The value of *σ* is chosen as a percentage of the deterministic forcing, hence $\sigma ={f}_{\sigma }{\bar{E}}_{a}$, based on observational data from *P* − *E* time series in the Atlantic ocean, assuming that the noise represents interannual time scale variability^26^. We estimated a lower bound of this amplitude *f*_*σ*_ to be 0.1 (see Supplementary Section D).

**Figure 3:**
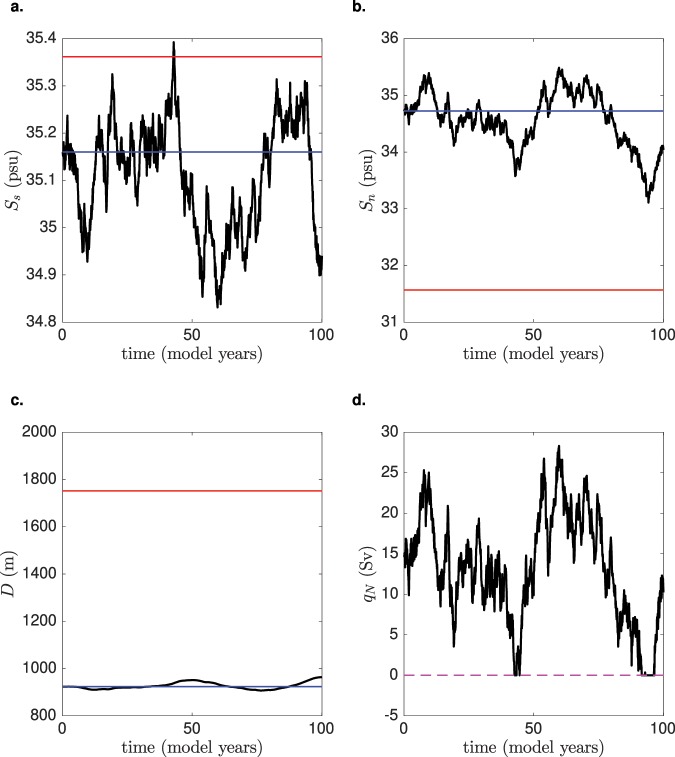
Trajectory of the box model, with reference parameters as in Supplementary Table S1, and with ${\bar{E}}_{a}=0.29$ Sv and *f*_*σ*_ = 0.16. The corresponding value of *M*_*ov*_ is −0.21 Sv. The initial condition is centered on the ‘on’ state of the system. Plots are shown for (**a**) *S*_*s*_, (**b**) *S*_*n*_, (**c**) *D* and (**d**) *q*_*N*_. The blue (red) line indicates the ‘on’ (‘off’) steady state, for the chosen parameters. The dashed magenta line indicates a zero transport.

In Fig. 3 an example of a model trajectory is shown, which is initialized at the ‘on’ AMOC equilibrium state (with ${\bar{E}}_{a}$ = 0.29 Sv and *M*_*ov*_ = −0.21 Sv). The applied noise in the freshwater forcing initially affects the polar boxes, and then propagates through the rest of the basin. The salinity in the southern box *S*_*s*_ (Fig. 3a) switches between the two equilibrium values (see Supplementary Section C), while the variability in the salinity of the northern box *S*_*n*_ (Fig. 3b) is not strong enough to reach the value corresponding to the ‘off’ AMOC state. Moreover, the depth of the pycnocline *D* (Fig. 3c) remains essentially constant throughout the whole simulation (*D* ~ 900 m in comparison to the 1700 m necessary to reach the value of the ‘off’ AMOC state). The most frequent abrupt changes involve the downwelling *q*_*N*_, which repeatedly decreases to zero throughout the duration of the simulation (Fig. 3d). However, the AMOC does not seem to undergo a full transition to the ‘off’ state, as shown by the behaviour of the other variables.

These results are very instructive on the behaviour of the system, as they indicate that the AMOC does not necessarily collapse (reach the ‘off’ state), even if the salinity in the boxes show quite some variability. In fact, the fast variations in the freshwater input determine large changes in the salinity of the polar boxes, to the point where the downwelling collapses. However, such collapse would have to be sustained for a long time to be able to considerably affect the deep ocean circulation. For this reason, we consider two kinds of transitions in the model: an F-type (fast) transition with a temporary cessation of the downwelling, and an S-type (slow) transition to an ‘off’ state. As already suggested by the model results in Fig. 3, probabilities for the two types of transitions substantially differ in magnitude.

Depending on the transition event one wants to study, the TAMS method has to be properly implemented, in order to select for the trajectories that undergo the transition. See Supplementary Section B for details on the choices made in this work.

**Figure 4:**
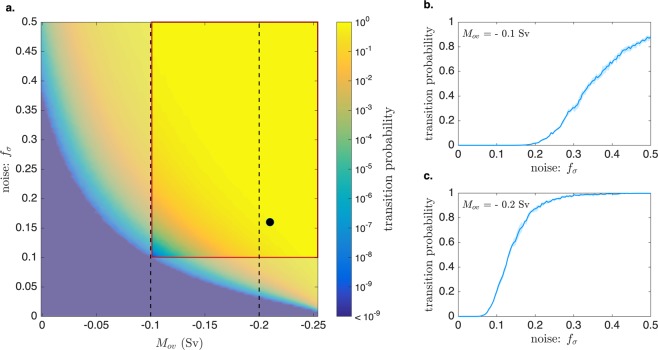
(**a**) Transition probabilities of the F-type transition (cessation of the downwelling) in 100 years, calculated for each couple of the parameters (*M*_*ov*_, *f*_*σ*_), chosen on a 100 × 125 grid. The computation was done with the TAMS algorithm, which was repeated 15 times for each transition probability computation. In this way, the error bars (not shown) turn out to be quite narrow (see panels (b,c) as an example). The area that corresponds to the range of parameters that can best represent the present-day climate is highlighted in the red box. It is bounded by the minimum amplitude of noise found in observations (0.1), and the observed range of values of *M*_*ov*_ (−0.35 to −0.1 Sv). The black circle indicates the parameters chosen for the time simulation in Fig. 3. (**b**,**c**) Transition probabilities as a function of *f*_*σ*_ for two particular choices of *M*_*ov*_ as indicated by the dashed lines in (**a**). The shaded areas represent the interquartile range for the probabilities. Notice the steepness of the gradient with respect to the value of the noise, which increases with more negative values of *M*_*ov*_.

To compute the transition probabilities for a range of possible values of freshwater forcing for F-type transitions, we ran 15 instances of the TAMS algorithm for a grid of size 100 × 125 in the $({\bar{E}}_{a},\sigma )$ space. Because we can connect ${\bar{E}}_{a}$ and *M*_*ov*_ through the steady state (Fig. 2d), and the noise is a fraction *f*_*σ*_ of the steady state value ${\bar{E}}_{a}$, we plot (Fig. 4a) the transition probabilities, using a transition time interval of 100 years, in the space (*M*_*ov*_, *f*_*σ*_).

The range of parameters, which corresponds to the present-day climate conditions, is the region bounded by *f*_*σ*_ > 0.1 and −0.35 Sv < *M*_*ov*_ < −0.1 Sv, taking the values at 24°S as representative for *M*_*ov*_^20^. The range of transition probabilities is then quite broad: depending on the actual value of *M*_*ov*_ and the stochastic forcing, an F-type transition can be very unlikely to occur (bottom-left area of the plot), or near certain (top-right area). Hence, we show the transition probability as a function of *f*_*σ*_ for *M*_*ov*_ = −0.1 Sv and *M*_*ov*_ = −0.2 Sv in Fig. 4b,c, respectively. For *M*_*ov*_ = −0.1 Sv the transition probabilities only become non-negligible for *f*_*σ*_ > 0.2, but for *M*_*ov*_ = −0.2 Sv (which could be seen as a mean value over the available estimates), the transition probability is already about 15% at *f*_*σ*_ = 0.1.

A sensitivity analysis, as shown in the Supplementary Section E, indicates that the computed probabilities are robust under reasonable changes in several of the parameters, in particular transport by the southern subtropical gyre *r*_*S*_, vertical diffusivity *κ* and eddy diffusivity *A*_*GM*_ (for which reference values were shown in Supplementary Table S1).

When determining the S-type transition probability of the AMOC to the ‘off’ state we obtain very low probabilities (<10^−9^) within 100 years, regardless of the values of the parameters of the model. If we increase the time interval, we find that only for time scales of the order of 10,000 years, the transition probabilities become non-negligible. An example of the evolution of the system on long time scales, showing an S-type transition, is shown in the Supplementary Section F.

## Summary and Discussion

In this paper we investigated the probability of noise-induced transitions of the AMOC to a collapsed state within a specific time period, using a conceptual box-model representation of the AMOC circulation^17^. Thanks to its simplicity, the model is suitable for the application of the Trajectory-Adaptive Multilevel Splitting (TAMS) algorithm. At the same time, it is quite comprehensive in terms of physical processes driving the circulation and allows to calculate the freshwater transported by the AMOC at 30°S (*M*_*ov*_), which connects the results of our model to state-of-art climate models and observations. In the model, the sign of *M*_*ov*_ represents a perfect indicator for the multiple equilibria regime (Fig. 2d), in line with what has been found in more detailed numerical models^8,11,15^.

The analysis of the time evolution of the stochastic model shows that two kinds of transitions occur, namely a cessation of the downwelling in the North Atlantic (called F-type transition) and a full transition to a collapsed state (called S-type transition). We found that, while an F-type transition can have very high transition probabilities over a period of 100 years under a reasonable choice of the parameters, the S-type transition seems unlikely to occur over this period, regardless of the values of the parameters involved. As expected, the probability of F-type transitions increases with decreasing *M*_*ov*_ (more negative) and with increasing noise amplitude *f*_*σ*_ (see Fig. 4a). For the area in the parameter space by which the present-day climate is best represented, our results indicate that the probability of a (temporary) cessation of the downwelling is almost certain for values of *M*_*ov*_ < −0.2 Sv.

Indeed, such transitions may have been found already in the RAPID program measurements^3,27^: the dips in the time series of the AMOC strength at 26.5°N (up to negative values) suggest the occurrence of extreme events in the circulation which are not directly connected to any subsequent collapse of the whole circulation system. They may be induced either by noise in the freshwater flux, in the heat flux or in the wind-stress field. The same phenomenon can be found in most of the CMIP5 models, where the time series of the AMOC at 26.5°N shows several dips during control simulations. The comparison between the occurrence of extremes in the transports associated with wind anomalies and dips in the AMOC strength suggests that the role of the stochastic buoyancy forcing can lead to extreme events in the AMOC. Indeed, while some extreme events in the AMOC can be attributed to anomalies in the wind field, others seem to occur independently of those anomalies. Therefore, we believe that anomalies in the freshwater forcing are responsible for (F-type) transitions in the AMOC (see the Supplementary Section G for more details).

In the context of paleoclimate transitions, such as the Dansgaard-Oeschger events, it is interesting that S-type transitions, involving a full-scale transition to an ‘off’ state, have only non-negligible transition probabilities in our model on multi-millennial time scales. However, to connect our model results to these events, a change in the background climate state (glacial) is necessary and possibly also a slow variation of the noise and the freshwater forcing should be introduced in the model, which is outside the scope of this paper.

## Supplementary information

Supplementary Information
