# Supplementary material for: Transition Probabilities of Noise-induced Transitions of the Atlantic Ocean Circulation
Source: Sci Rep. 2019 Dec 30;9:20284. doi: 10.1038/s41598-019-56435-6 (PMC6937345; doi:10.1038/s41598-019-56435-6)
Supplement: Supplementary file 1 — Supplementary Information [file 41598_2019_56435_MOESM1_ESM.pdf]

# Supporting Information for "Transition Probabilities of Noise-induced Transitions of the Atlantic Ocean Circulation"

**Daniele Castellana[1,*], Sven Baars[2], Fred W. Wubs[2], and Henk A. Dijkstra[1,3]**

[1]Institute for Marine and Atmospheric research Utrecht, Department of Physics, Utrecht University, Utrecht, The Netherlands.
[2]Bernoulli Institute for Mathematics, Computer Science and Artificial Intelligence, University of Groningen, Groningen, The Netherlands.
[3]Centre for Complex Systems Studies, Department of Physics, Utrecht University, Utrecht, The Netherlands.
[*]d.castellana@uu.nl

## ABSTRACT

The Atlantic Meridional Overturning Circulation (AMOC) is considered to be a tipping element of the climate system. As it cannot be excluded that the AMOC is in a multiple regime, transitions can occur due to atmospheric noise between the present-day state and a weaker AMOC state. For the first time, we here determine estimates of the transition probability of noise-induced transitions of the AMOC, within a certain time period, using a methodology from large deviation theory. We find that there are two types of transitions, with a partial or full collapse of the AMOC, having different transition probabilities. For the present-day state, we estimate the transition probability of the partial collapse over the next 100 years to be about 15%, with a high sensitivity of this probability to the surface freshwater noise amplitude.

## Contents

1. Section A: Box model
2. Section B: Details of TAMS
3. Section C: Additional Bifurcation diagrams
4. Section D: Analysis of Freshwater noise
5. Section E: Sensitivity Analysis for F-type transitions
6. Section F: Result for S-type transitions
7. Section G: F-type transitions in a control simulation of a GCM

## Section A: Box model

The equations governing the evolution of the system read:

$$
\begin{aligned}
\frac{\mathrm{d}\left(V_t S_t\right)}{\mathrm{d}t} &= q_S(\theta(q_S)S_{ts} + \theta(-q_S)S_t) + q_U S_d - \theta(q_N)q_N S_t + r_S(S_{ts} - S_t) \\
&\quad + r_N(S_n - S_t) + 2E_s S_0, \\
\frac{\mathrm{d}\left(V_{ts} S_{ts}\right)}{\mathrm{d}t} &= q_{Ek} S_s - q_e S_{ts} - q_S(\theta(q_S)S_{ts} + \theta(-q_S)S_t) + r_S(S_t - S_{ts}), \\
\frac{\mathrm{d}\left(V_n S_n\right)}{\mathrm{d}t} &= \theta(q_N)q_N(S_t - S_n) + r_N(S_t - S_n) - (E_s + E_a)S_0, \\
\frac{\mathrm{d}\left(V_s S_s\right)}{\mathrm{d}t} &= q_S(\theta(q_S)S_d + \theta(-q_S)S_s) + q_e S_{ts} - q_{Ek} S_s - (E_s - E_a)S_0, \\
\left(A + \frac{L_{xA} L_y}{2}\right)\frac{\mathrm{d}D}{\mathrm{d}t} &= q_U + q_{Ek} - q_e - \theta(q_N)q_N, \\
S_0 V_0 &= V_n S_n + V_d S_d + V_t S_t + V_{ts} S_{ts} + V_s S_s,
\end{aligned}
\tag{S1}
$$

where the function $\theta(x)$ is a step function, which is 1 for a positive argument and 0 otherwise. Through this function, we can represent the different circulation given by the different sign of $q_S$ and by the possibility that the downwelling ($q_N$) is zero. The last equation expresses the conservation of salinity in the basin. The transports depend, in turn, on the variables, via the following relations[1]:

$$
\begin{aligned}
q_{Ek} &= \frac{\tau L_{xS}}{\rho_0 |f_S|}, \\
q_e &= A_{GM} \frac{L_{xA}}{L_y} D, \\
q_U &= \frac{\kappa A}{D}, \\
q_N &= \eta \frac{\rho_n - \rho_{ts}}{\rho_0} D^2, \\
q_S &= q_{Ek} - q_e,
\end{aligned}
\tag{S2}
$$

where the density of the generic box $i$ is defined as

$$
\rho_i = \rho_0 \left(1 - \alpha(T_i - T_0) + \beta(S_i - S_0)\right). \tag{S3}
$$

The equation for the downwelling, introduced by *Cimatoribus et al.*[1], is based on the northward geostrophic transport above the thermocline in the high latitudes. Thus, $q_N$ is determined by the density contrasts between the northern part and southern part of the Atlantic Ocean. The relation was found in several ocean GCM studies[2–4], even with near eddy-resolving resolution. For instance, Fig. 8 in[2] shows the linear dependence of the NADW (North Atlantic Deep Water) flow on the density difference between two latitudinal strips (50-55°N and 35-40°S) at mid-depth (750 m). Moreover, this linear dependence was successfully tested in[5].

Also the volume of the pycnocline, as well as the one of the deep box, depend on the state of the system, in particular on the variable $D$:

$$
\begin{aligned}
V_t &= AD, \\
V_{ts} &= \frac{L_{xA} L_y}{2} D, \\
V_d &= V_0 - V_n - V_s - V_t - V_{ts}.
\end{aligned}
\tag{S4}
$$

The reference parameter values, together with their descriptions, are shown in table S1.

| Parameters used in the model | | |
|---|---|---|
| $V_0$ | $3 \times 10^{17}$ m$^3$ | total volume of the basin |
| $V_n$ | $3 \times 10^{15}$ m$^3$ | volume of the northern box |
| $V_s$ | $9 \times 10^{15}$ m$^3$ | volume of the southern box |
| $A$ | $1 \times 10^{14}$ m$^2$ | horizontal area of the Atlantic pycnocline |
| $L_{xA}$ | $1 \times 10^{7}$ m | zonal extent of the Atlantic Ocean at its southern end |
| $L_y$ | $1 \times 10^{6}$ m | meridional extent of the frontal region of the Southern Ocean |
| $L_{xS}$ | $3 \times 10^{7}$ m | zonal extent of the Southern Ocean |
| $\tau$ | 0.1 N m$^{-2}$ | average zonal wind stress amplitude |
| $A_{GM}$ | 1700 m$^2$s$^{-1}$ | eddy diffusivity |
| $f_S$ | $-10^{-4}$ m$^3$ | Coriolis parameter |
| $\rho_0$ | 1027.5 kg m$^{-3}$ | reference density |
| $\kappa$ | $10^{-5}$ m$^2$s$^{-1}$ | vertical diffusivity |
| $S_0$ | 35 psu | reference salinity |
| $T_0$ | 5 K | reference temperature |
| $T_n$ | 5 K | temperature of the northern box |
| $T_{ts}$ | 10 K | temperature of the box *ts* |
| $\eta$ | $3 \times 10^{4}$ m s$^{-1}$ | hydraulic constant |
| $\alpha$ | $2 \times 10^{-4}$ K$^{-1}$ | thermal expansion coefficient |
| $\beta$ | $8 \times 10^{-4}$ psu$^{-1}$ | haline contraction coefficient |
| $r_S$ | $1 \times 10^{7}$ m$^3$s$^{-1}$ | transport by the southern subtropical gyre |
| $r_N$ | $5 \times 10^{6}$ m$^3$s$^{-1}$ | transport by the northern subtropical gyre |
| $E_s$ | $0.17 \times 10^{6}$ m$^3$s$^{-1}$ | symmetric freshwater flux |

**Table S1.** Reference parameters used in equations (S1) - (S4).

## Section B: Details of TAMS

Consider a system of SDAEs (Stochastic Differential Algebraic Equations), and assume that the corresponding deterministic system has two steady states, A and B, for a certain choice of the parameters. Several methods are available in order to find transition probabilities between A and B. In case the corresponding deterministic system can be described in terms of a potential, the transition rate for transitions induced by white additive noise is given by the Eyring-Kramers formula[6,7]. This result, later extended to non-gradient systems[8], is derived under very strict assumptions on the noise, which are often not valid in real physical systems. Furthermore, the quantity we are interested in is the probability that the system undergoes a transition within a certain time, which is not always related to the transition rate, especially if the noise that forces the system is large. The Trajectory-Adaptive Multilevel Splitting algorithm[9] is based on the idea of simulating a large ensemble of trajectories, and discarding the ones that do not reach B and splitting (or branching) trajectories that are closer to B. As a consequence, the probability that a trajectory reaches B keeps increasing, which is why this method is more efficient than a brute-force method.

At each time step, the trajectories are ranked according to a so-called reaction coordinate. The ones with the lowest value of the reaction coordinate are discarded, while new ones are generated, by randomly branching other trajectories. A certain weight $w_i$, which is related to the number of discarded trajectories at each time iteration $i$, is a measure of the probability of a trajectory to reach the next step. An unbiased estimator of the transition probability $\hat{p}$ can be obtained multiplying these weights:

$$\hat{p} = \frac{N_{\text{trans}}}{N} \prod_{i=0}^{k} w_i, \tag{S5}$$

where $k$ is the number of iterations of the algorithm, $N_{\text{trans}}$ is the number of trajectories that eventually reached the destination equilibrium and $N$ is the total number of trajectories in the ensemble.

In our results in section 3 of the paper, we used 1000 trajectories for each repetition of the algorithm, which ensures an adequate precision of the results. A step-by-step description of the algorithm is given in[9] with the details on the statistical properties of the estimator of the probability presented in[10].

The reaction coordinate gives a measure of how close to the state B - and far from the state A - each trajectory goes. Depending on the event one wants to study, an appropriate function has to be chosen.

For an F-type transition, we define the reaction coordinate in TAMS as a linear function of the downwelling ($q_N$), which assumes the value 0 when the system is in the 'on' state and 1 when $q_N = 0$, hence

$$\phi_F(\mathbf{x}) = 1 - \frac{q_N(\mathbf{x})}{q_N(\mathbf{x}_{on})}, \tag{S6}$$

where $\mathbf{x}$ represents the state vector of the system, $\mathbf{x}_{on}$ the 'on' state, and $q_N(\mathbf{x})$ the value of the downwelling.

For an S-type transition, i.e., a full transition to the 'off' state, the reaction coordinate in TAMS is taken as

$$\phi_S(\mathbf{x}) = \eta - \eta e^{-4\frac{\|\mathbf{x}-\mathbf{x}_{on}\|_2^2}{\|\mathbf{x}_{off}-\mathbf{x}_{on}\|_2^2}} + (1-\eta) e^{-4\frac{\|\mathbf{x}-\mathbf{x}_{off}\|_2^2}{\|\mathbf{x}_{off}-\mathbf{x}_{on}\|_2^2}}, \tag{S7}$$

where $\eta = \|\mathbf{x}_u - \mathbf{x}_{on}\|_2 / \|\mathbf{x}_{off} - \mathbf{x}_{on}\|_2$ is the normalized distance between the unstable steady state $\mathbf{x}_u$ and the stable steady state $\mathbf{x}_{on}$.

As an example of the use of the reaction coordinates, consider the system of SDEs

$$\begin{aligned} \mathrm{d}x_1(t) &= \left(x_1(t) - x_1(t)^3\right)\mathrm{d}t + \sigma_1\,\mathrm{d}W_1(t) \\ \mathrm{d}x_2(t) &= -2x_2(t)\,\mathrm{d}t + \sigma_2\,\mathrm{d}W_2(t), \end{aligned} \tag{S8}$$

where $\mathbf{x} = (x_1, x_2)$ is the state vector, the constants $\sigma_1$ and $\sigma_2$ represent the amplitudes of the noise and $W_1, W_2$ two independent Wiener processes. The deterministic part of the equations describes a gradient system, where the double-well potential reads

$$V(x_1, x_2) = \frac{1}{4}x_1^4 - \frac{1}{2}x_1^2 + x_2^2. \tag{S9}$$

Among the three steady states of the system, $\mathbf{x}_A = (-1, 0)$ and $\mathbf{x}_B = (1, 0)$ are stable, while $\mathbf{x}_C = (0, 0)$ is unstable. In order to find transition probabilities between the two equilibria with the TAMS algorithm, within a certain time $T$, the reaction coordinate is chosen according to equation (S7), where $\mathbf{x}_{on}$, $\mathbf{x}_{off}$ and $\mathbf{x}_u$ correspond respectively to $\mathbf{x}_A$, $\mathbf{x}_B$ and $\mathbf{x}_C$. An example of iteration of the algorithm, based on the ranking given by the reaction coordinate, is shown in Fig. S1. The process is iterated, using a sufficient amount of trajectories (usually at least 1000), for a certain number of times, and the transition probability is calculated according to equation (S5).

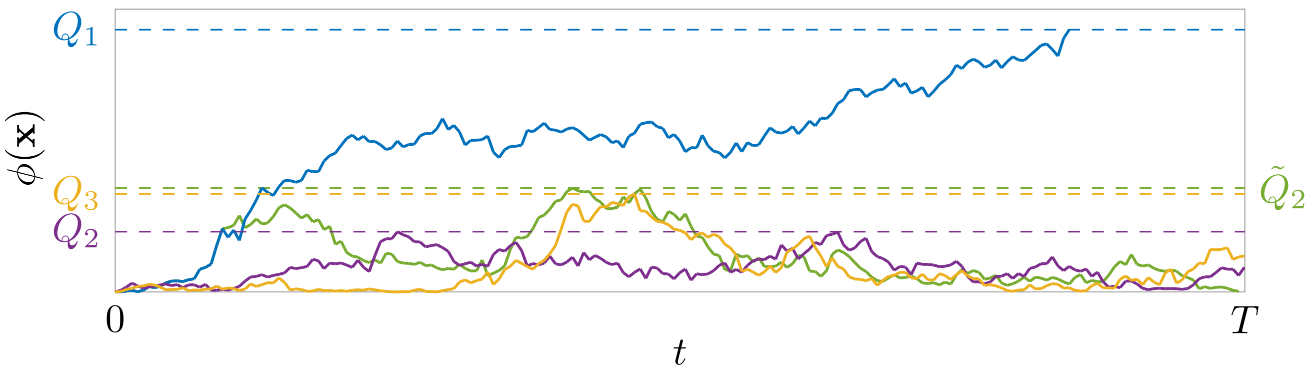


**Figure S1.** Example iteration of TAMS for the double-well gradient system (equations (S8)), using an ensemble of three trajectories. The trajectories (yellow, blue and purple) are initialised at $\mathbf{x}_A$, simulated for a certain amount of time T, and then ranked according to the reaction coordinate (S7). As the purple trajectory has the lowest maximum value of the reaction coordinate ($Q_2$), it is discarded and a new trajectory (green) is generated, branching randomly from one of the remaining trajectories (in this case, the blue one). This new trajectory has a new maximum value of the reaction coordinate ($\tilde{Q}_2$) which is, in this case, larger than $Q_2$.

## Section C: Additional Bifurcation diagrams

The steady states for the other variables of the model (complementary to the ones in the main text) are shown here.

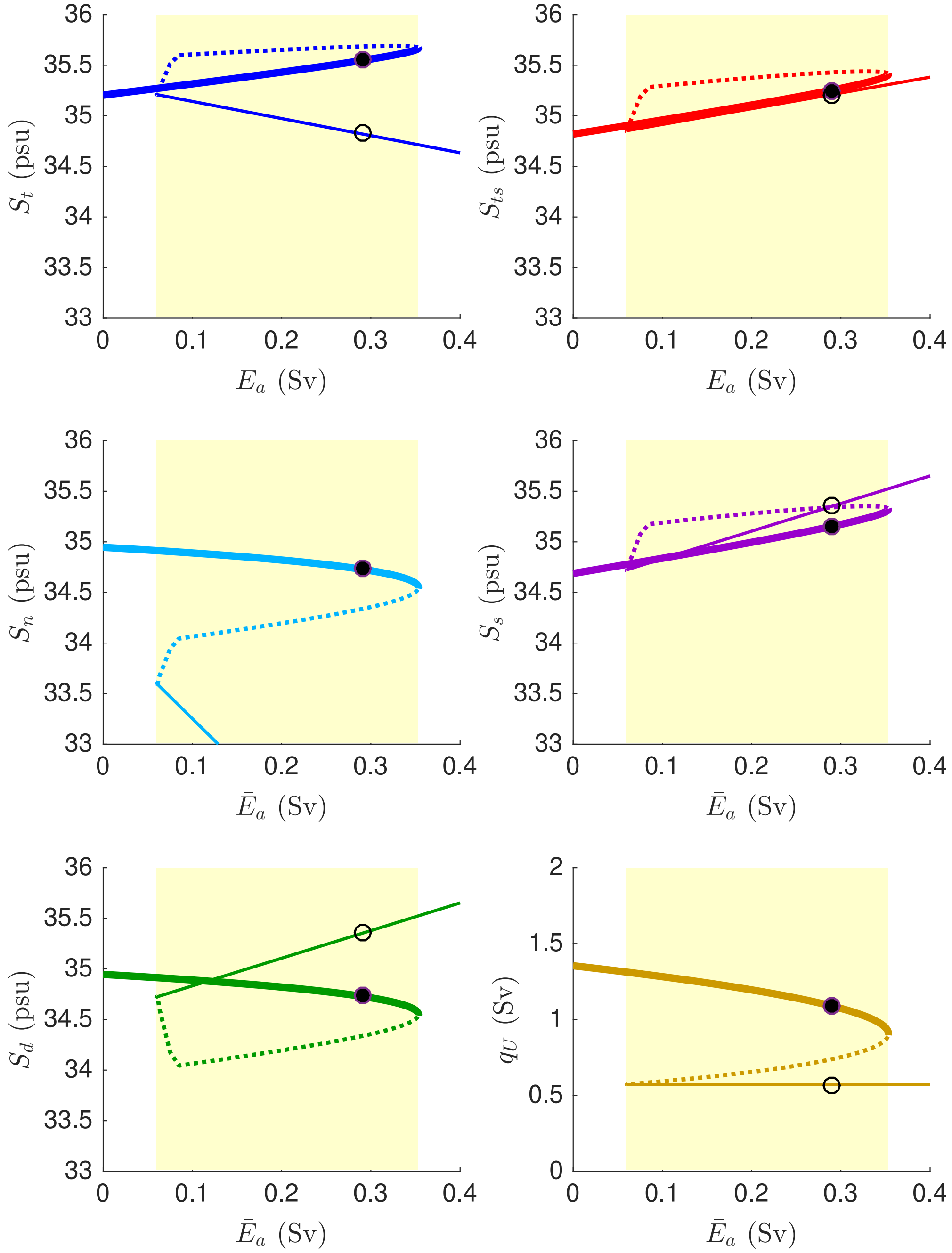


**Figure S2.** Bifurcation diagrams for the salinity variables of the model and for the diffusive upwelling ($q_U$) with respect to the deterministic parameter $\bar{E}_a$. Solid (dashed) lines indicate stable (unstable) equilibria of the system. Among the solid lines, the thicker ones represent the so called AMOC 'on' state, while the thinner ones correspond to the collapsed 'off' state. The yellow area indicates the bistable regime. The black circles indicate the value of $\bar{E}_a$ chosen for the time simulation in Fig. 3 in the main text. The starting (destination) equilibrium belongs to the 'on' ('off') branch of the diagram.

## Section D: Analysis of Freshwater noise

The amplitude of the noise forcing our system was estimated from observational data relative to the freshwater forcing in the Atlantic Ocean. The values the P-E (precipitation minus evaporation) were year-averaged and integrated over two basins, corresponding to the northern and southern boxes in the model. The time series thus obtained were summed and subtracted from each other, resulting respectively in the symmetric and antisymmetric component of the freshwater forcing, namely $E_s$ and $E_a$ in the model (see Fig. S3). The value of $f_\sigma$ is then computed as the ratio between the standard deviation and the mean value of $E_a$. Such value is representative of the interannual time scale variability of the forcing. If more high-frequency noise is used, ocean mixed layer processes (not represented in the box model) will integrate this noise to give interannual variability in the surface salinity affecting the north-south density gradient. Hence, in our box model these interannual variations are an adequate description of unresolved processes. As a first order approximation of the process, we assumed the noise to be white and normally distributed. In[11], a similar choice was made, given that the decorrelation time of the stochastic freshwater forcing, computed by their model, is one year. We used $f_\sigma$ as the lower bound of the noise amplitude, as, if smaller time scales are considered, the associated variability increases.

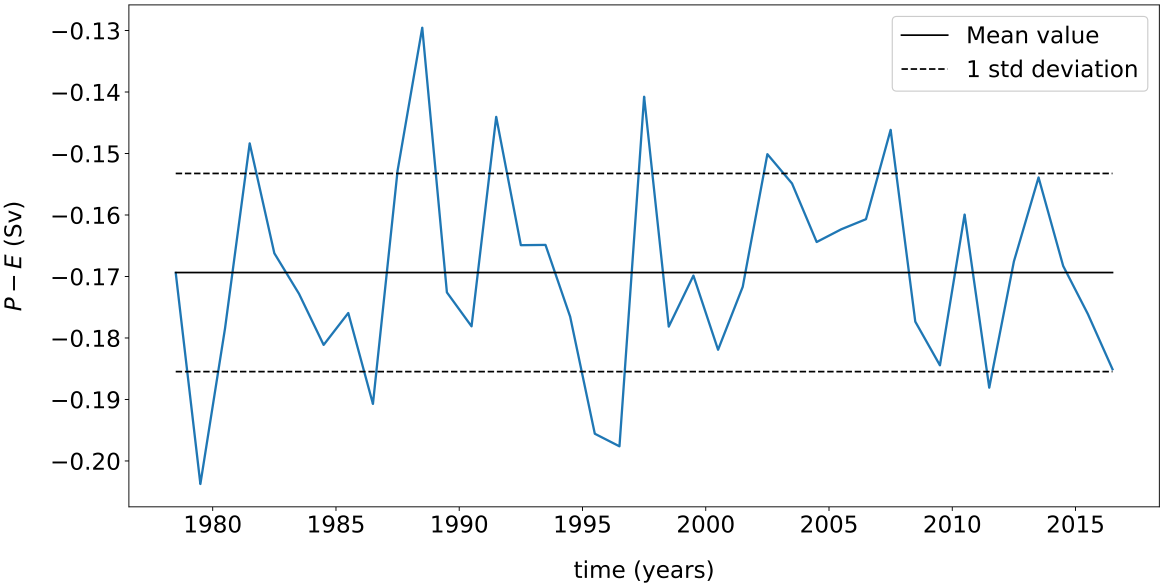


**Figure S3.** From the observed yearly averaged P-E in the Atlantic Ocean, from ERA-Interim Archive at ECMWF[12], its antisymmetric part is shown, respect to the North (50°N - 70°N and South (South of 40°S) Atlantic. The black horizontal lines indicate the mean value (solid line) and one standard deviation confidence interval (dashed lines). The value of $f_\sigma$, that is the ratio between the standard deviation and the mean value, is about 0.1.

## Section E: Sensitivity Analysis for F-type transitions

In order to test the robustness of our results, we computed transition probabilities for slightly different versions of our model, characterised by different values of certain parameters. In all the simulations, we kept the value of $M_{ov}$ and the noise constant. Note that, as the configuration of the system varies, a certain value of $M_{ov}$ does not correspond to a unique value of $\bar{E}_a$. Based on the results of the analysis, shown in Fig. S4, we can conclude that the method seems to be robust under uncertainties in the estimation of the parameters.

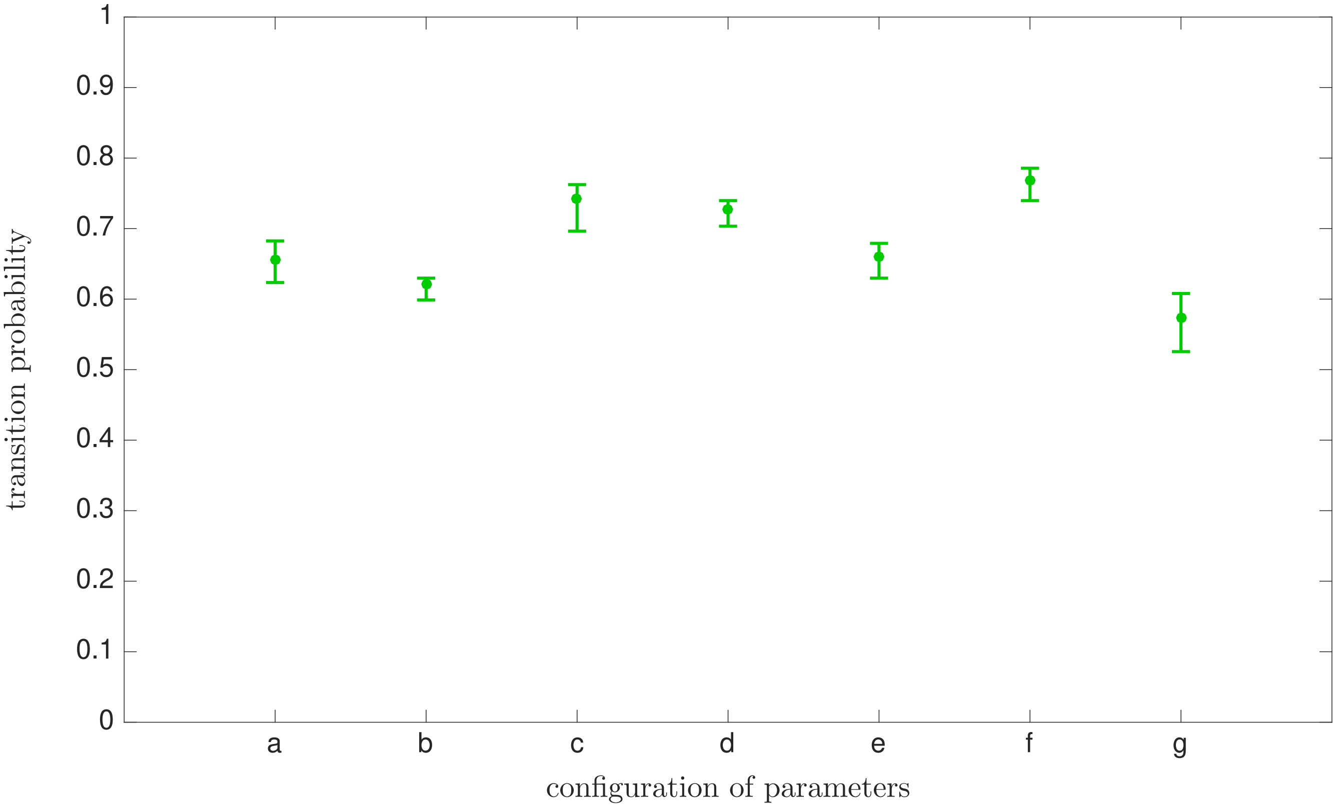


**Figure S4.** Transition probabilities calculated for $T = 100$ years, $M_{ov} = -0.18$ Sv and $f_\sigma = 0.18$, for different choices of the parameters $r_S$ (transport by the southern subtropical gyre), $\kappa$ (vertical diffusivity) and $A_{GM}$ (eddy diffusivity). For each value of the probability, the bar represents the interquartile range, calculated from 10 different repetitions of the TAMS algorithm. The configuration (a) is the standard one, according to the parameters in table S1. Configurations (b) and (c) are characterised by a different value of $r_S$, respectively $1.25 \times 10^7$ and $0.75 \times 10^7$ $m^3s^{-1}$ ($\pm$ 25%). In configurations (d) and (e), $\kappa$ was set respectively to $2 \times 10^{-5}$ and $0.5 \times 10^{-5}$ $m^2s^{-1}$ (2 times and half the value of the standard configuration). Finally, in configurations (f) and (g) $A_{GM}$ was set respectively to 1870 and 1530 $m^2s^{-1}$ ($\pm$ 10%).

## Section F: Result for S-type transitions

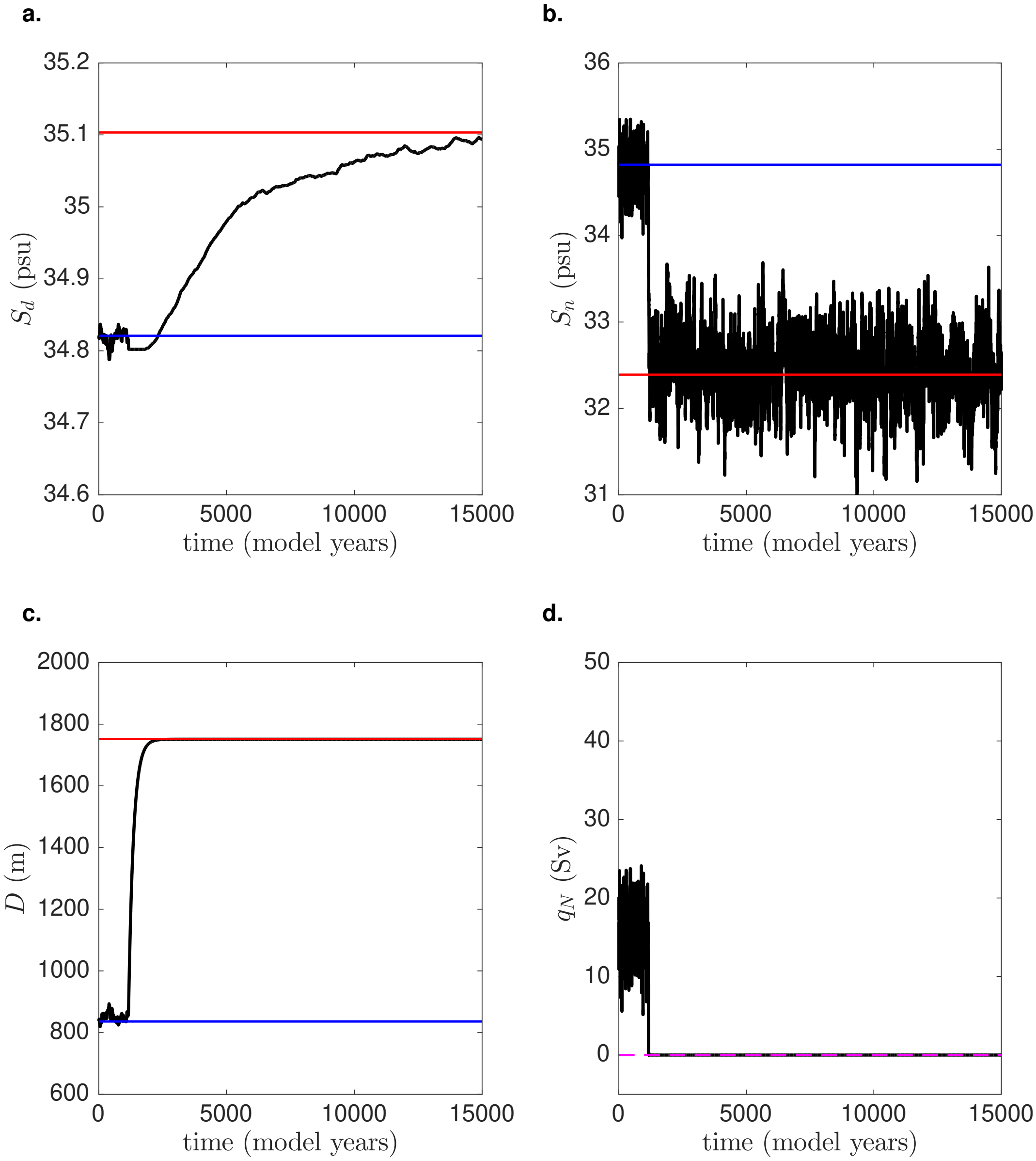


**Figure S5.** Trajectory of the model (S1) on a long time scale (15,000 years), with reference parameters as in Table S1 and with $\bar{E}_a = 0.20$ Sv and $f_\sigma = 0.16$. The corresponding value of $M_{ov}$ is $-0.12$ Sv. The initial condition is centered on the 'on' state of the system. Plots are shown for (a) $S_d$, (b) $S_n$, (c) $D$ and (d) $q_N$. The blue (red) line indicates the 'on' ('off') state, for the chosen parameters. The plot (d) shows the evolution of the downwelling in the North Atlantic. The dashed magenta line indicates null transport. The figure clearly shows that the different quantities reach the values corresponding to the collapsed state at different times. The system undergoes a full transition at the end of the time frame considered here, as illustrated by the value of the salinity of the deep box, the slowest variable, which eventually reaches the value corresponding to the 'off' state. The downwelling had already stopped after about 1000 years, indicating that an F-type transition had occurred much earlier.

## Section G: F-type transitions in a control simulation of a GCM

The AMOC varies over a wide range of time scales and such variations can be driven by buoyancy or wind forcing anomalies. In[13] a detailed analysis of the observed AMOC variability is performed, with particular attention to the anomaly in 2009/2010. It is argued that the extreme event was caused by westerly wind anomalies associated with the North Atlantic Oscillation (NAO) negative phase, which was found to be correlated with the surface wind-driven Ekman transport.

When looking at the strength of the AMOC in pre-industrial control simulations of CMIP5 models, we can observe several extreme events in the circulation. In this section we show results from the CMIP5 model MIROC5[14]. The value of $M_{ov}^{35°S}$ for this model was calculated as -0.036 Sv[15], which indicates that the AMOC is in a multiple equilibria regime. Therefore, buoyancy-induced extreme events might occur in the circulation: with the terminology introduced in this paper, such events may be associated with the occurrence of F-type transitions. In Fig. S6, the time series of the maximum value of the AMOC, the zonally integrated Ekman transport and the maximum Sverdrup transport in the western part of the basin, at 26.5°N, are shown. The comparison between the time series shows that some dips in strength of the AMOC are most likely related to the occurrence of wind anomalies, while others may be the consequence of changes in the buoyancy forcing.

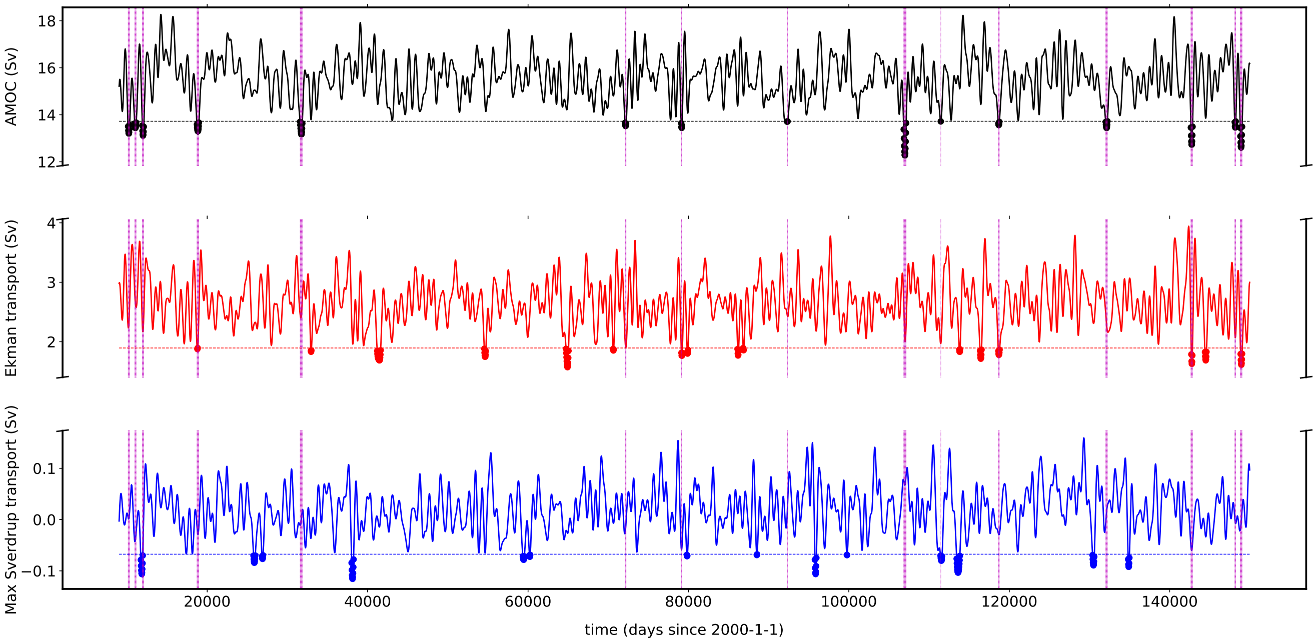


**Figure S6.** 20 month Butterworth low-pass filtered time series of the maximum value of the AMOC at 26.5°N (black), the zonally integrated Ekman transport (red) and the maximum Sverdrup transport at the same latitude (blue), in the pre-industrial control simulation of the CMIP5 model MIROC5. The dashed horizontal lines indicate, for each time series, the lower bound of the 2 standard deviations confidence interval around the mean. The circles identify the time points at which extreme events occur, defined as the values extending below the confidence interval. The dotted magenta vertical lines are drawn in correspondence with the extreme events of the AMOC on top of all the time series. In this way, it is possible to assess whether a certain event is related with anomalies in the wind circulation. Some of the events seem to occur independently of changes in both Ekman and Sverdrup transports (e.g. the one found between time 80000 and 100000).

## References


1. Cimatoribus, A. A., Drijfhout, S. S. & Dijkstra, H. A. Meridional overturning circulation: Stability and ocean feedbacks in a box model. *Clim. Dyn.* **42**, 311–328, DOI: https://doi.org/10.1007/s00382-012-1576-9 (2012).
2. Rahmstorf, S. On the freshwater forcing and transport of the Atlantic thermohaline circulation. *Clim. Dyn.* **12**, 799–811, DOI: https://doi.org/10.1007/s003820050144 (1996).
3. Weijer, W., Maltrud, M. E., Hecht, M. W., Dijkstra, H. A. & Kliphuis, M. A. Response of the Atlantic Ocean circulation to Greenland Ice Sheet melting in a strongly-eddying ocean model. *Geophys. Res. Lett.* **39**, DOI: https://doi.org/10.1029/2012GL051611 (2012).
4. Toom, M. d. *et al.* Response of a Strongly Eddying Global Ocean to North Atlantic Freshwater Perturbations. *J. Phys. Oceanogr.* **44**, 464–481, DOI: https://doi.org/10.1175/JPO-D-12-0155.1 (2014).
5. Levermann, A. & Fürst, J. J. Atlantic pycnocline theory scrutinized using a coupled climate model. *Geophys. Res. Lett.* **37**, DOI: https://doi.org/10.1029/2010GL044180 (2010).
6. Eyring, H. The activated complex in chemical reactions. *The J. Chem. Phys.* **3**, 107–115, DOI: https://doi.org/10.1063/1.1749604 (1935).
7. Kramers, H. Brownian motion in a field of force and the diffusion model of chemical reactions. *Physica* **7**, 284–304, DOI: https://doi.org/10.1016/s0031-8914(40)90098-2 (1940).
8. Bouchet, F. & Reygner, J. Generalisation of the Eyring–Kramers transition rate formula to irreversible diffusion processes. *Annales Henri Poincaré* **17**, 3499–3532, DOI: https://doi.org/10.1007/s00023-016-0507-4 (2016).
9. Lestang, T., Ragone, F., Bréhier, C.-E., Herbert, C. & Bouchet, F. Computing return times or return periods with rare event algorithms. *J. Stat. Mech. Theory Exp.* 043213, DOI: https://doi.org/10.1088/1742-5468/aab856 (2018).
10. Rolland, J. & Simonnet, E. Statistical behaviour of adaptive multilevel splitting algorithms in simple models. *J. Comput. Phys.* **283**, 541–558, DOI: https://doi.org/10.1016/j.jcp.2014.12.009 (2015).
11. Ganopolski, A. & Rahmstorf, S. Abrupt glacial climate changes due to stochastic resonance. *Phys. Rev. Lett.* **88**, DOI: https://doi.org/10.1103/physrevlett.88.038501 (2002).
12. Dee, D. P. *et al.* The ERA-interim reanalysis: Configuration and performance of the data assimilation system. *Q. J. Royal Meteorol. Soc.* **137**, 553–597, DOI: https://doi.org/10.1002/qj.828 (2011).
13. Zhao, J. & Johns, W. Wind-forced interannual variability of the atlantic meridional overturning circulation at 26.5°n. *J. Geophys. Res. Ocean.* **119**, 2403–2419, DOI: https://doi.org/10.1002/2013JC009407 (2014).
14. Taylor, K. E., Stouffer, R. J. & Meehl, G. A. An overview of CMIP5 and the experiment design. *Bull. Am. Meteorol. Soc.* **93**, 485–498, DOI: https://doi.org/10.1175/BAMS-D-11-00094.1 (2012).
15. Mecking, J. V., Drijfhout, S. S., Jackson, L. C. & Andrews, M. B. The effect of model bias on atlantic freshwater transport and implications for amoc bi-stability. *Tellus A: Dyn. Meteorol. Oceanogr.* **69**, 1299910, DOI: https://doi.org/10.1080/16000870.2017.1299910 (2017).
